# Role of Home-Modification Training for Care Managers

**DOI:** 10.1093/geroni/igab046.2875

**Published:** 2021-12-17

**Authors:** Akiko Nishino, Yoritaka Harazono, Moeko Tanaka, Kazunori Yoshida, Toko Funaki, Ryosuke Takada, Takenori NASU, Taketo TOBIMATSU

**Affiliations:** 1 Former The University of Tokyo, bunkyo-ku, Tokyo, Japan; 2 The University of Tokyo, bunkyo-ku, Tokyo, Japan

## Abstract

With the aging of society, the long-term care insurance system -which includes home modifications to continue living at home- was established in 2000. However, the quality of home modifications has been persistent issue, and effective training is expected to conclusively solve this problem. To this end, the purpose of this study is to clarify the rational for training care managers who plan home modifications. A survey comprising two sets of questionnaires was conducted; one set encompassed is all 62municipalities in Tokyo, whereas the other involved care manager who participated in training program. The results of the first questionnaire showed that, out of 62 municipalities, 9 (14.5%) provided training on home modification, of which 8 (88.9%) provided training on administrative procedures. In one municipality that provided training on practical aspects of home modification, we provide questionnaires to 59 care managers participating in the training. -Lectures on administrative procedures, physical conditions of invalids, and reading drawings were conducted by administrative staff, occupational therapists, and architects, respectively. Afterwards, the participants attended a planning workshop. According to the questionnaire conducted after the workshop, 80.4% of the participants could understand home modifications in the system, 85.5% understood how to modify homes based on the occupants’ symptoms and physical conditions, 81.6% could interpret drawings, 90.2% could plan modifications, and 81.6% found the training useful. These findings indicate that the training of care managers has indeed been effective in actual practice. Improving the quality of home modifications through multidisciplinary cooperation is significant in maintaining home life.

